# Flexural Behavior of Slender UHPC Prestressed Beams Without Passive Reinforcement

**DOI:** 10.3390/ma19101936

**Published:** 2026-05-08

**Authors:** Juan Navarro-Gregori, Yeiner A. Gómez-Velásquez, Juan A. Mateu-Sánchez, Pedro Serna, José R. Martí-Vargas

**Affiliations:** Institute of Concrete Science and Technology (ICITECH), Universitat Politècnica de València, Camino de Vera s/n, Building 4N, 46022 Valencia, Spainjuamasa8@cam.upv.es (J.A.M.-S.); pserna@cst.upv.es (P.S.); jrmarti@upv.es (J.R.M.-V.)

**Keywords:** effective prestress, flexural behavior, prestressed beams, prestress losses, slender precast elements, UHPC

## Abstract

This study examines the flexural behavior of slender ultra-high-performance fiber-reinforced concrete (UHPC) beams with cross-sections intended for scalable precast production. The members are prestressed only, with no passive reinforcement. An experimental program on eighteen beams combined three cross-sectional typologies (rectangular as a reference, I-shaped, and H-shaped), three UHPC mixes with fiber contents of 130, 160, and hybrid 130 + 60 kg/m^3^, and two prestressing layouts (bottom-only and symmetric top-and-bottom). Prestress was indirectly controlled by evaluating effective tendon stress, with time-dependent prestress losses quantified using vibrating-wire strain gauges. Four-point bending tests provided material characterization and structural response, enabling assessment of stiffness and ultimate capacity. The results highlight the coupled influence of cross-section, fiber dosage, and prestress configuration on global response. Post-cracking residual strength in UHPC promoted stable multiple cracking, while prestressing governed deflection control. Residual equivalent flexural tensile stresses above 35 MPa at deflections over 50 mm, span/70, were achieved in I- and H-shaped sections, exceeding those of rectangular sections. Overall, the study substantiates the feasibility of lightweight, durable, prestressed UHPC members that deliver significant self-weight reductions without compromising reliability.

## 1. Introduction

Ultra-high-performance concrete (UHPC) may be regarded as an advanced evolution of fiber-reinforced cementitious composites, capable of combining very high compressive strength, significant post-cracking tensile resistance, and outstanding durability. These attributes make UHPC particularly attractive for structural members in which reduced self-weight, slender geometries, long service life, and prefabrication efficiency are key design drivers. Over the last two decades, its use has progressively expanded from local applications, such as joints, overlays, and rehabilitation works, to primary load-bearing members and bridge components [1,2,3,4,5,6].

Although the term UHPC is defined in different ways in the international literature and in current technical documents, and also referred to as UHPFRC (Ultra-high-performance fiber-reinforced concrete), there is broad agreement that this family of materials combines a dense and optimized particle packing, a very low water-to-binder ratio, the absence of coarse aggregate or the use of very fine granular skeletons, and the incorporation of steel fibers in amounts sufficient to provide a significant post-cracking tensile response. Design-oriented references such as the French specifications and recommendations [7], the Japanese JSCE guidelines [8], and the ACI 239 report [9] consistently highlight three key features of UHPC: very high compressive strength, enhanced tensile behavior due to fiber reinforcement, and exceptional durability.

From a structural and sustainability perspective, UHPC presents a clear paradox. On the one hand, it requires high Portland cement content and carefully selected constituents, which can raise concerns about cost and embodied carbon. On the other hand, its superior mechanical performance and durability open the possibility of radically reducing material consumption at the structural level by developing lighter, thinner, and longer-lasting members. Therefore, the feasibility and sustainability of UHPC are closely linked to the design of highly optimized structural solutions in which the total amount of concrete—and, consequently, the global cement consumption—remains below that of equivalent conventional concrete solutions [1,4].

Within this framework, the combination of UHPC with prestressing offers a particularly promising route to the development of lightweight, highly slender precast elements. Prestressing improves serviceability by controlling tensile stresses and deflections, while UHPC provides high compressive strength, tensile resistance after cracking, and enhanced durability. This synergy can enable thinner webs, more efficient flange arrangements, lower self-weight, and potentially longer spans. It is especially attractive for industrialized construction and precast production, where reduced member weight may also facilitate transport, handling, erection, and, in some applications, improved seismic performance owing to lower structural mass [1,10,11].

Despite these advantages, the design of prestressed UHPC members still involves important unresolved issues. Among the most relevant are the quantification of prestress losses, the role of the tensile response of UHPC at service and ultimate states, and the identification of structural configurations capable of combining high strength with adequate residual response and ductility. Recent studies have shown that prestressed UHPC members may fail shortly after crack localization if reinforcement rupture governs the response, whereas more favorable structural behavior can be achieved when the member is proportioned to promote strain redistribution and delayed localization. In addition, predicting time-dependent prestress losses remains a critical challenge, as creep and shrinkage in UHPC differ from those in conventional concrete [12,13,14,15,16,17].

Another key issue concerns the tensile characterization of UHPC and its representativeness at the member level. The tensile and flexural response of UHPC is strongly influenced by fiber type, fiber content, fiber–matrix bond, and especially fiber distribution and orientation. These variables depend on the casting procedure, rheology, mold geometry, and member slenderness. Consequently, tensile properties derived from standard prismatic specimens should often be regarded as indicative when extrapolated to real structural members, particularly in thin-walled or highly slender elements in which the production process may significantly modify fiber alignment and, therefore, structural performance [18,19,20].

The effect of restrained shrinkage must also be carefully considered in UHPC structural members. Because shrinkage strains may be higher than those observed in conventional concrete, the interaction with passive reinforcement or prestressing tendons can induce non-negligible tensile stresses in the matrix even before external loading is applied. In prestressed concrete members, this phenomenon directly affects the effective prestress state and may alter the onset of cracking, service response, and long-term performance. These aspects are especially relevant in slender elements, in which restraint effects and time-dependent deformations may have a proportionally greater structural impact [12,18].

The broader challenge, therefore, is not simply to use a high-performance material, but to define viable and robust structural concepts that can fully exploit it. This perspective has already been highlighted in previous research and in practical applications, including the first UHPC footbridge built in Spain [21] and durability-oriented developments such as the concept of ultra-high durability concrete [22]. However, whereas bridge applications have led most of the international development of UHPC, there is still considerable room for progress in precast building and industrialized construction, where the benefits of weight reduction, durability, and geometric refinement could be particularly significant [1,21,23].

Against this background, the present study investigates the response of slender prestressed UHPC beams without passive reinforcement, conceived as lightweight members for scalable precast production. Three cross-sectional typologies were considered, namely a rectangular section taken as reference, an I-shaped section, and an H-shaped section. The main variables were the UHPC mixture and the prestressing layout, consisting of bottom-only prestressing and symmetric prestressing at the top and bottom faces. In addition, the UHPC mixtures were characterized in compression and by unnotched four-point bending tests, while tendon stress was monitored from prestressing to bending testing to quantify the effective prestress state prior to loading.

The objectives of this study were: (i) to quantify the evolution of tendon stress and the prestress losses occurring between transfer and bending testing; (ii) to assess the flexural response of slender prestressed UHPC beams without passive reinforcement as a function of cross-sectional typology, UHPC mixture, and prestressing layout; and (iii) to identify the most efficient sectional concept in terms of load-carrying capacity, residual response, and material efficiency for potential application in lightweight precast members. The novelty of the study lies in combining prestress monitoring with flexural testing, thereby enabling a consistent interpretation of structural efficiency based on the actual effective prestress state at the time of testing. This provides a basis for maximizing the structural potential of slender prestressed UHPC members in practical applications. Finally, it should be noted that a detailed analysis of the cracking process under service conditions is beyond the scope of this paper.

## 2. Experimental Program

### 2.1. Test Specimens

**Figure 1:**
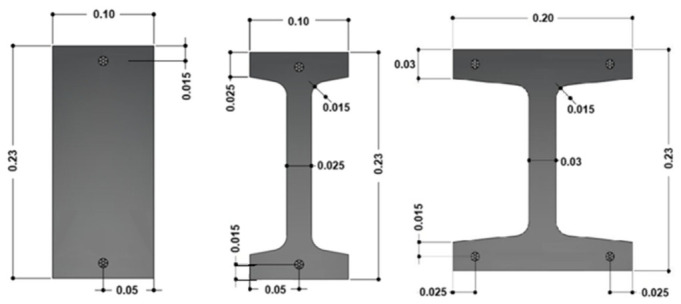
Cross-section details of the prestressed UHPC beams (Units in m).

The experimental program consisted of eighteen prestressed UHPC beams arranged into three series of six specimens, each series with a different cross-section geometry: one rectangular (S0), one I-shaped (S1), and one H-shaped (S2). Within each series, the parameters investigated were the UHPC type—defined by three steel fiber contents (C1: hybrid 130 + 60 kg/m^3^; C2: 160 kg/m^3^; and C3: 130 kg/m^3^)—and two prestressing layouts (T-type: bottom-only; and D-type: symmetric top-and-bottom). It is important to note that none of the beams included longitudinal or transverse passive reinforcement, not even in the end regions near the tendon anchorages. Figure 1 and Table 1 present the detailed geometries of the prestressed UHPC cross sections. Each beam was assigned an ID following the notation X_YZ, where X denotes the cross-section type (S0, S1, or S2), Y the UHPC type (C1, C2, or C3), and Z the prestressing layout (T or D).

**Figure 2:**
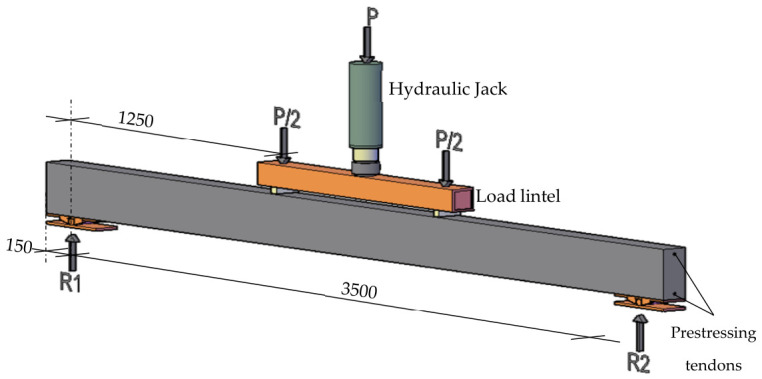
UHPC beams test setup (Units in mm).

The total length of each beam is 3800 mm to allow a four-point bending test (Figure 2), providing a clear span between support axes of 3500 mm and a distance between loading points of 1000 mm. Consequently, the resulting shear slenderness is a/d = 1250 mm/215 mm = 5.81, which ensures a flexure-dominated response.

### 2.2. Material Properties

Portland cement CEM I 42.5R SR5, silica fume, two types of fine aggregate, water, and a superplasticizer were used to prepare the UHPC matrix. Table 2 shows the dosage adopted in the mixtures for each type of concrete: C1 (hybrid 130 + 60 kg/m^3^), C2 (160 kg/m^3^), and C3 (130 kg/m^3^). The water-to-cement ratio (*w*/*c*) was 0.21. The mixtures were designed to be self-compacting, and to achieve this, a superplasticizer was added at a dosage of approximately 0.5% by cement weight. The mix design aimed to obtain a characteristic compressive strength of 135 MPa on 100 mm cubes.

Two types of steel fibers were used: (i) smooth, straight steel fibers (Bekaert (Zwevegem, Belgium) Dramix^®^ OL 13/0.20) and (ii) hooked-end steel fibers (Bekaert (Zwevegem, Belgium) Dramix^®^ 80/30 BGP), the latter used only in mix C1. The hybrid mix C1, including longer fibers, was used to evaluate its potential positive effect on the residual response of the UHPFRC beams. Table 3 summarizes the mechanical and physical properties of the steel fibers used in this experimental program. All UHPC batches were produced at the facilities of the Institute of Concrete Science and Technology (ICITECH), and a total of ten distinct batches were prepared. In each batch, an S0 and an S1 beam were cast with identical concrete mix designs and prestressing tendon configurations. For the S2 beams, the target was to cast pairs with the same concrete but different prestressing schemes; however, two batches deviated from this plan. As a result, six S2 beams were cast across four batches. The fibers were gradually incorporated into the mixture to ensure proper dispersion within the concrete and to guarantee the desired self-compactability.

The UHPC compressive strength was determined using 100 mm cubes, in accordance with the European standard UNE-EN 12390-3:2020 [24]. Increased loading was applied at a rate of 0.6 MPa/s. A total of 112 cubes were tested along the ten batches, and specimens were tested at 2, 7, 28, and 90 days in most of the batches to investigate the time evolution of concrete compressive strength.

The tensile behavior of the UHPC was assessed through unnotched four-point bending tests (4PBTs) performed on 100 × 100 × 450 mm prismatic specimens. A total of 44 specimens were tested in accordance with the Spanish standard UNE 83519:2024 [25] to obtain the UHPC tensile properties. The sample size comprised 22 specimens for concrete type C2 and 11 specimens for concrete types C1 and C3.

The prestressing tendons were 7-wire steel strands Y 1860 S7 9.3 (ultimate tensile strength of 1860 MPa, and nominal diameter of 9.30 mm) supplied by the Portuguese manufacturer SOCITREL, with a mass per unit length of 0.409 kg/m, corresponding to an equivalent steel area of 52.1 mm^2^.

### 2.3. Prestressing Setup

**Figure 3:**
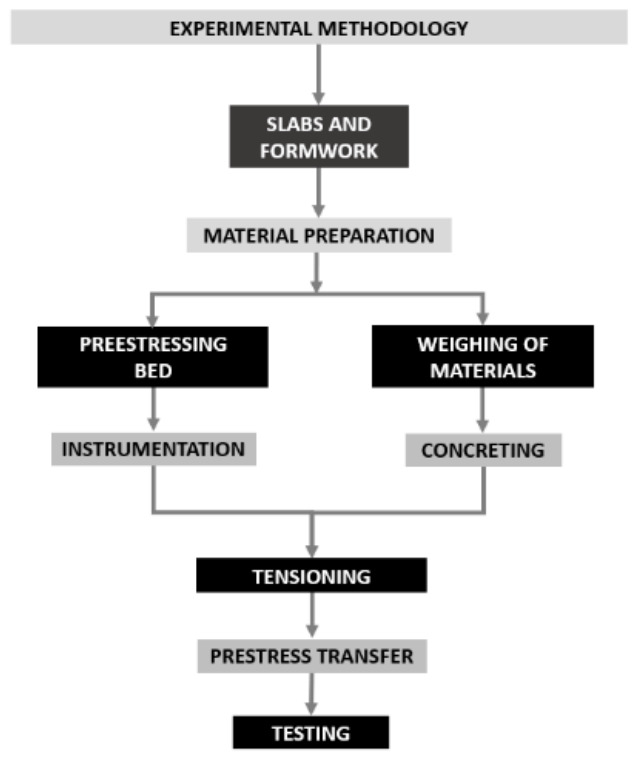
Workflow of the prestressing experimental methodology.

Figure 3 provides a schematic overview of the adopted procedure, summarizing the experimental methodology used to fabricate the prestressed UHPC beams.

**Figure 4:**
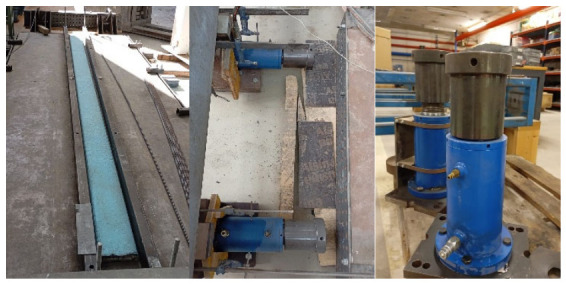
Slab, formwork, and hydraulic jacks.

A 1600 mm × 4340 mm steel formwork panel was mounted on supports, providing a constant elevation of 70 mm above the laboratory strong floor. For beams S1 (I-shaped) and S2 (H-shaped), the cross-section geometry was defined using plastic-coated expanded polystyrene (EPS) molds (Figure 4). The side forms were completed with two steel stringers on each side, installed with a clear inner spacing of 230 mm, since the beam concreting process was carried out with the beams considered rotated 90° relative to the orientation of their cross-section, shown in Figure 1. This arrangement ensured geometric uniformity and facilitated subsequent handling and prestressing operations.

The constituents employed to produce the UHPC mix across the different batches are detailed in Section 2.2. Product quality control was verified for use in concrete production in the ICITECH lab.

The prestressing bed setup (Figure 4) consisted of four reaction abutments and two perforated steel beams incorporating through-holes at uniform 50 mm spacing. These steel beams were mounted horizontally at both ends of the formwork and fulfilled a dual structural function: (i) establishing the prescribed strands profile and (ii) providing the bearing surface for the anchorage devices. One steel beam was rigidly restrained to serve as the passive anchorage, whereas the opposite steel beam was displaced using two hydraulic jacks equipped with mechanical locking systems. Additionally, between each of the two hydraulic jacks and the perforated steel beam, an HBM C6A 500 kN force transducer was installed, thereby enabling the controlled, measurable application of the prestressing force.

After the precise weighing of all constituent materials and the positioning of the prestressing strands in their designated locations, steel strain gauges were installed on the strands to record stress variations throughout the prestressing operation. In addition, vibrating-wire strain gauges (VWSGs) were placed at the mid-plane of the cross-section, at the mid-span of each beam, to monitor the time-dependent concrete deformations.

The configuration of the prestressing system and the magnitude of the applied force varied depending on the cross-section geometry. The strands were tensioned to 75% of their ultimate tensile capacity, corresponding to a stress level of 1395 MPa and generating a total prestressing force of 75 kN per strand.

**Figure 5:**
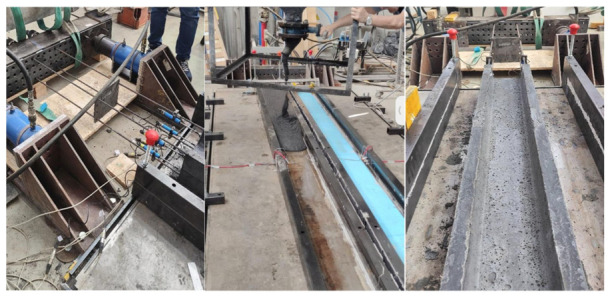
Prestressing, casting, and transfer process.

A continuous, controlled casting sequence was applied, commencing at one end of the mold and progressing toward the opposite end to ensure homogeneous cross-section filling (Figure 5). In the cases of the S0 and S1 series, the UHPC was initially placed to a height of 7.3 cm and 14.1 cm, respectively. Subsequently, the upper EPS mold was positioned to define the desired section geometry while minimizing concrete overflow. Moreover, the cube and prismatic specimens were prepared.

The prestress transfer to the concrete was carried out 48 h after casting, once the UHPC had achieved sufficient compressive strength to resist the stresses induced by strand release. After prestress transfer, the beams were stored for a period of 3 months before the beam test setup.

### 2.4. Beam Test Setup

**Figure 6:**
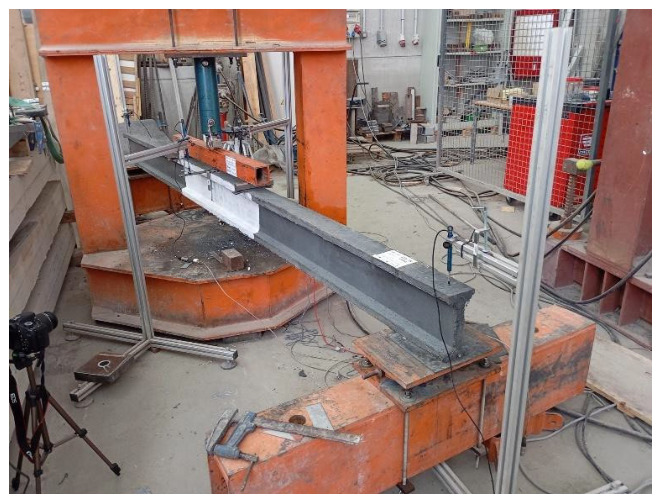
Beam test setup and instrumentation.

The test setup to perform the experiment is shown in Figure 2 and Figure 6. A 200 kN servo- hydraulic jack was used, which was connected to a universal testing machine. A force transducer HBM C6A 500 kN was placed between the steel lintel and the beam to register the load applied accurately. To measure displacements, four potentiometric displacement transducers were used, as shown in Figure 6. Two of them were used to measure the mid-span deflection—primarily to identify any potential rotations of the beam—while an additional transducer was placed at each support to capture undesired vertical movements and to determine the effective mid-span deflection. The test was recorded with a high-resolution digital camera to track the evolution of the crack pattern and, in particular, to document the specimen failure. In addition, two strain gauges were installed at mid-span, on the top and bottom surfaces of the beam, to monitor the evolution of sectional strains and to enable the experimental determination of the bending-moment–curvature response.

## 3. Test Results and Observation

The reported results include: (i) the outcomes of the UHPC characterization tests, (ii) the monitoring of strand stresses from pre-tensioning up to the start of the bending tests, and (iii) the bending-test response referenced to a well-defined initial stress state established by the monitored prestress levels.

### 3.1. UHPC Characterization Tests

For prestressed concrete specimens, it is essential to determine the compressive strength achieved by the concrete not only under service and ultimate conditions, but also at the time of prestress transfer, which in the present experimental program took place two days after casting. Table 4 summarizes the average concrete compressive strength results obtained at 2, 7, 28 days, and 90 days after casting; the coefficients of variation (%) are given in brackets. Compressive strength data at 2, 7 days, and 90 days were unavailable for some batches due to laboratory constraints, without affecting the reliability of the results.

**Figure 7:**
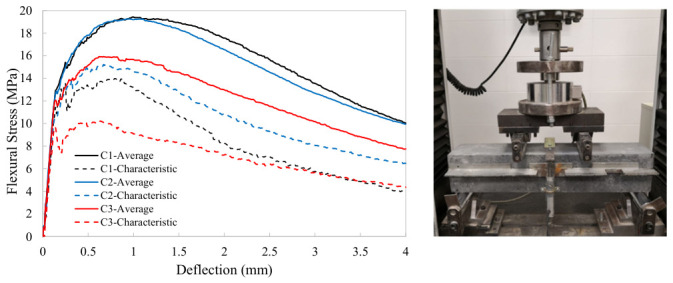
Average and characteristic four-point bending test results.

The results of the unnotched four-point bending tests (4PBTs) performed on 100 × 100 × 450 mm prismatic specimens are presented in Figure 7 as an initial assessment of the tensile behavior of the three UHPC types considered in this study. These tensile properties should be regarded as indicative only, since the tensile response measured in prismatic specimens may differ significantly from that developed in the UHPC prestressed beams. This is primarily due to differences in casting procedure, which may substantially affect fiber orientation and distribution. Nevertheless, the tests provide a useful basis for evaluating UHPC tensile performance until a more precise relationship between fiber distribution and orientation in prismatic specimens and prestressed UHPC beams is established.

Figure 7 shows the average and characteristic load–deflection curves obtained from the unnotched 4PBTs for the three concrete types C1, C2, and C3. The reported values correspond to results from different batches produced with the same concrete type. Among the three concrete types, C1 showed the best overall performance, although its average response was similar to that of C2, despite the latter containing 60 kg/m^3^ less fiber. This indicates that combining two fiber types in C1 did not lead to a significant overall improvement, except for an enhanced post-peak response. By contrast, C3 exhibited the worst performance.

### 3.2. Prestress Losses

Table 5 summarizes, for each beam, the total prestressing force measured immediately before transfer (*P*_1_) and immediately before the bending test (*P*_2_). The difference Δ*P* = *P*_1_ *− P*_2_ represents the combined prestress losses due to elastic shortening at transfer and the time-dependent prestress losses accrued during the curing/storage period prior to testing. The table also reports *P*_1_ and *P*_2_ as percentages of the strand ultimate tensile strength (*f_pu_* = 1860 MPa), along with the relative prestress loss Δ*P*/*P*_1_ (in %). It is important to note that the target prestressing force per strand was 75 kN, and the applied force was adjusted to this setpoint as closely as practicable.

**Figure 8:**
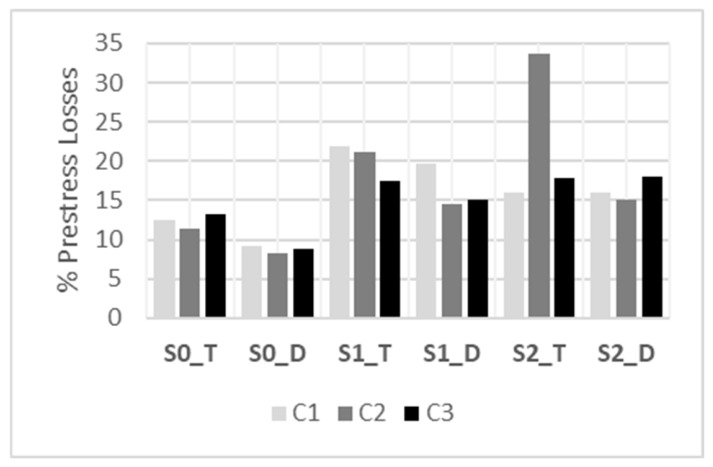
Prestress losses (Δ*P*/*P*_1_, %) by cross-section type and prestressing layout.

Complementing Table 5, Figure 8 summarizes the estimated prestress loss ratio, Δ*P*/*P*_1_ (%), grouped by cross-section type (S0, S1, S2) and prestressing configuration (T, D). This representation enables direct comparison across design alternatives and highlights the influence of both the cross-section geometry and the strand layout on the magnitude of prestress losses accrued between transfer and testing.

### 3.3. Bending Tests

**Figure 9:**
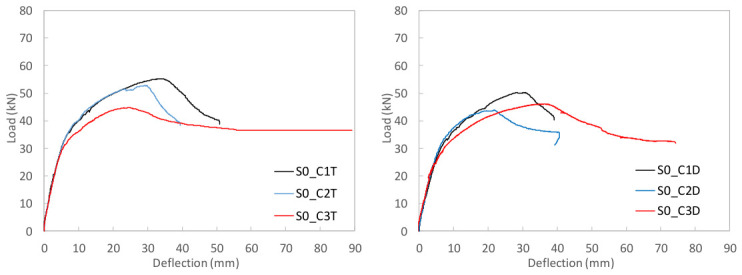
Load–mid-span deflection (P–δ) curves for the rectangular S0 series.

**Figure 10:**
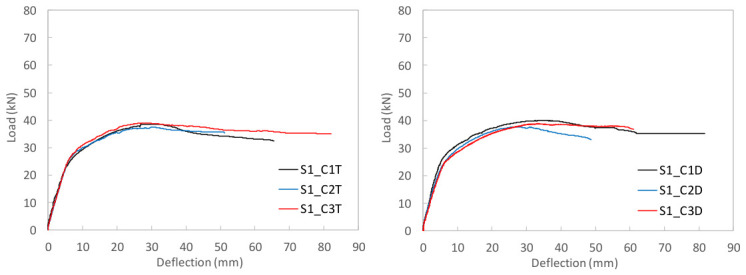
Load–mid-span deflection (P–δ) curves for the I-shaped S1 series.

**Figure 11:**
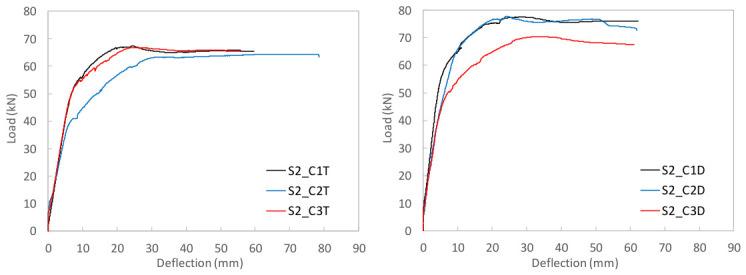
Load–mid-span deflection curves for the H-shaped S2 series.

**Figure 12:**
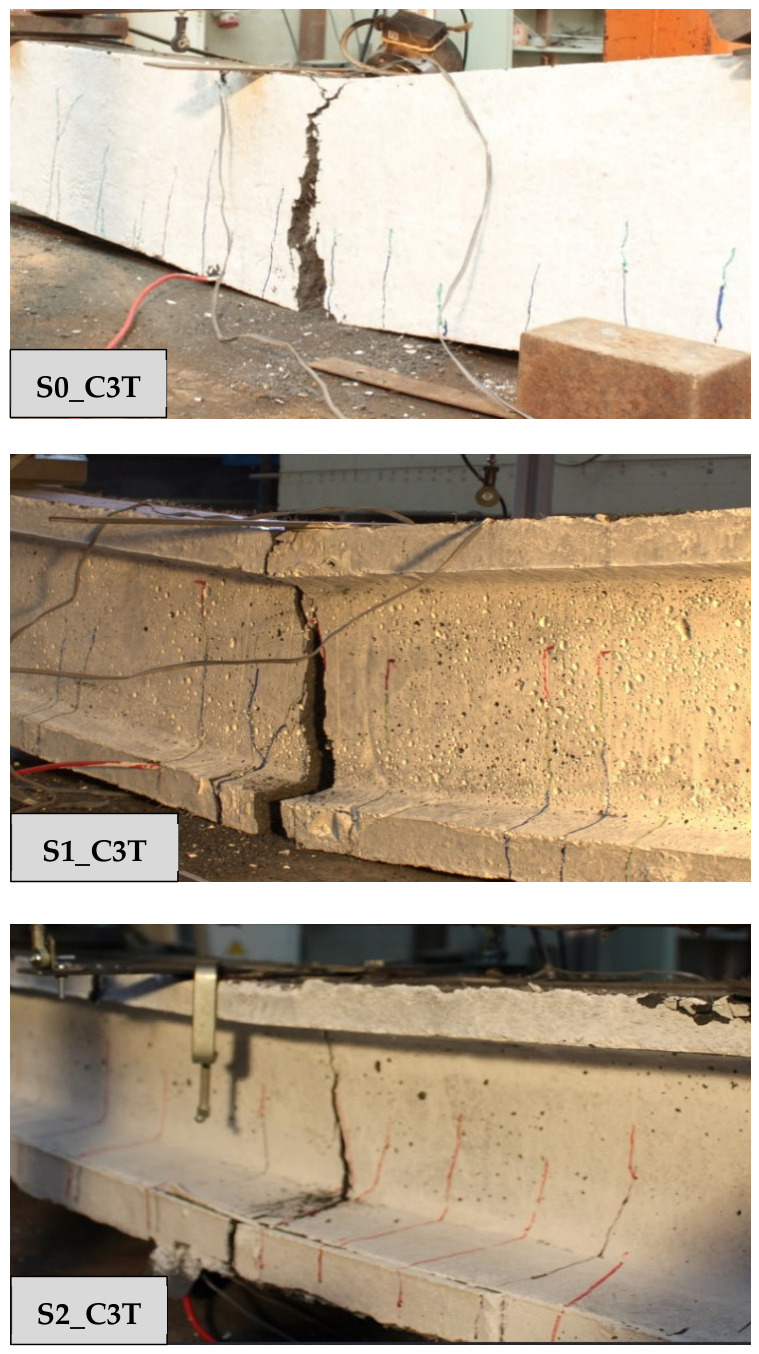
Representative tested beams at failure.

To study the flexural behavior of the UHPC beams, Table 6 reports, for each tested specimen, the ultimate load, *P_u_*, attained during the test, the corresponding deflection, *δ_u_*, and the maximum deflection recorded, *δ_max_*, as well as, the prestressing axial and bending moment forces, *N_p_* and *M_p_*, and the stress in the prestressing reinforcement at the onset of testing are provided. Moreover, Figure 9, Figure 10 and Figure 11 present the load–mid-span deflection (*P*–*δ*) curves for the eighteen tested beams, grouped by cross-section type. In each figure, the left panel shows the response of the three beams with a bottom-only prestressing layout, whereas the right panel shows the response with a symmetric prestressing layout on both faces. Within each panel, the three curves correspond to beams with the same cross-section and prestressing scheme, cast with the three different UHPC mixtures. Figure 12 shows, for illustrative purposes, the beams S0_C3T, S1_C3T, and S2_C3T at failure.

## 4. Discussion

The discussion of the results is organized into two subsections. The first focuses on the monitoring of strand stresses from pre-tensioning to the start of the bending tests. The second analysis shows the flexural response of the beams with respect to a well-defined initial stress state determined from the monitored prestress levels.

### 4.1. Prestress Losses

The prestress losses evaluated in Section 3 include both the elastic-shortening losses at transfer and the time-dependent prestress losses accumulated during storage up to the start of the bending tests. As shown in Table 5 and Figure 8, the rectangular S0 sections exhibited the lowest prestress losses. Within this series, the beams with symmetric prestressing on both faces (D-type) showed the smallest values, slightly below 10% and around 8%, whereas the S0 beams prestressed only at the bottom face (T-type) exhibited prestress losses between 11.46% and 13.18%. This can be attributed mainly to the higher concrete stress at the strand level in the T-type configuration compared with the D-type configuration, despite the higher total prestressing force applied in the latter. This trend was consistently observed for concretes C1, C2, and C3.

For the S1 series, the prestress losses were again higher in the T-type configuration (17.46–21.92%) than in the D-type configuration (14.52–19.65%). In all cases, the prestress losses in S1 were significantly higher than those in the rectangular S0 series, by about 5 percentage points on average. For the S2 series, prestress loss values ranged from 15.12% to 18.01%, with no significant difference between single-face and symmetric prestressing. An exception was beam S2_C2T, which exhibited an anomalously high prestress loss of 33.63%. This result is attributed to insufficient concrete maturity at transfer, since prestress was applied when the concrete compressive strength was still low (67.3 MPa average, see Table 4) and, consequently, the UHPC cracking strength. Under these conditions, tensile stresses likely developed in the upper layer of the section, possibly leading to microcracking. The tensile stress predicted at transfer for this beam was −4.46 MPa based on the prestressing force reported in Table 5 and the application of the Navier–Bernoulli equation assuming an uncracked cross-section, which was likely beyond the tensile capacity of the upper layer in the S2 section. The concrete type (C1, C2, or C3) appears to be less influential on the recorded prestress losses than the cross-sectional geometry and the prestressing layout within the section.

**Figure 13:**
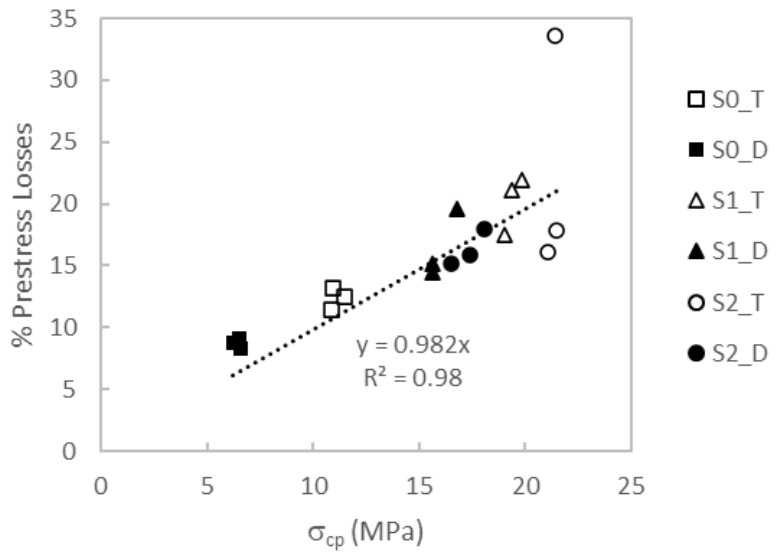
Relationship between prestress losses and compressive concrete stress.

To further support these experimental observations, Figure 13 compares the prestress-loss percentage measured in each beam with the concrete stress at the strand level, *σ_cp_*. A proportional relationship is observed in Figure 13, with % prestress losses increasing as the stress *σ_cp_* increases (beam S2_C2T excluded). The anomalous behavior of beam S2_C2T is again evident. This result underlines the need to transfer prestress at a stage that prevents cracking-induced reductions in sectional flexural stiffness, thereby avoiding excessive prestress losses, while still enabling the tensile strength of the concrete to be effectively considered in structural design. Prestress transfer must therefore be defined on the basis of a compromise between safety and efficiency.

### 4.2. Bending Tests

#### 4.2.1. Rectangular S0 Series

Figure 9 shows the characteristic shape of the load–deflection curves in the rectangular S0 series. The response begins with a linear branch associated with the uncracked state, followed by a gradual reduction in flexural stiffness as microcracking initiates at the tension face of the beam. This degradation continues up to the maximum load, after which a descending post-peak branch develops towards a residual load-carrying capacity. The residual response reflects the remaining contribution of the prestressing strand at the tension face, whereas the peak load marks the maximum activation of the steel fibers or the point of fibers’ maximum efficiency. Beyond the peak, the critical crack localizes, and progressive fiber pull-out occurs at a critical section located at an arbitrary position within the constant-moment region between the two loading points (Figure 2).

Marked differences are observed among the beams cast with the three concrete types. For beams prestressed only at the bottom face, C1 performed best, followed by C2 and C3. In the S0 series with symmetric prestressing, beam S0_C3D showed poorer service behavior but ultimately exceeded the failure load of its counterpart S0_C2D. This suggests that the casting procedure may significantly influence flexural behavior. In particular, the rectangular section appears highly sensitive to fiber distribution and orientation under the specific manufacturing conditions considered. Overall, except for beam S0_C3D, the S0_T beams reached higher maximum loads than the S0_D beams.

It should also be noted that no crushing of the compression chord was observed in any of the six beams of the S0 series, which indicates that the compressive strength of the UHPC was not attained at peak load. This suggests that the section could be prestressed with a higher force or optimized with a smaller cross-section, thereby reducing material consumption and better exploiting the concrete compressive capacity. In some specimens, the test was continued beyond the residual branch, leading to the development of a major crack in the tension zone. The crack opening increased to the point that yielding of the bottom strand, and eventually fracture of the strand, occurred after a pronounced residual ductility response. For safety reasons, some beams were not tested up to this failure stage. Notably, no shear damage was observed in any of the tested beams, even though no transverse reinforcement was used.

#### 4.2.2. I-Shaped S1 Series

Figure 10 shows the characteristic load–deflection response of the I-shaped S1 series. Overall, the behavior is similar to that of the S0 series, but with a much less pronounced peak load. The response consists of an initial uncracked linear branch, followed by a cracked branch with a gradual increase up to the maximum load, and a post-peak residual branch controlled by the bottom prestressing reinforcement yielding. Unlike the S0 series, the maximum load variation among the three concrete types is within approximately 6%, indicating that the influence of fiber content and type is secondary compared to the effect of cross-sectional geometry. This suggests that, although fiber distribution and orientation may vary from beam to beam, the fiber volume does not significantly affect either the peak load or the residual flexural response.

The residual loads are similar to those observed in the corresponding S0 series, resulting in a highly ductile response, albeit with lower peak loads than those of S0. Although this is unfavorable in terms of strength, it is consistent with the use of a lighter cross-section. The maximum loads in the S1_T series ranged from 37.50 to 38.93 kN, slightly below those of the S1_D series, which ranged from 37.74 to 40.02 kN These values should be interpreted together with the different initial prestress states reported in Table 6. As in the S0 series, all beams exhibited a pronounced residual ductility branch. No crushing of the compression chord was observed, and fracture of the bottom strand occurred only at large deflections, approximately between 50 and 80 mm (Table 6). In some tests, failure was not completed due to laboratory safety concerns.

#### 4.2.3. H-Shaped S2 Series

As previously noted in Section 3.3, the production of the beams with the largest cross-section, i.e., the H-shaped S2 series, involved some difficulties during casting when the top mold was installed. This operation generated internal pressure in the fresh concrete, which in turn induced significant lateral thrust on the steel side formwork. In some specimens, deformation of these steel side panels increased the overall depth of the beam at mid-span, i.e., in the region where the bending test was later conducted. The measured effective depth, d, to the lower prestressing strand was as follows for the six S2 beams: S2_T series (S2_C1T, d = 217 mm; S2_C2T, d = 213 mm; S2_C3T, d = 215 mm) and S2_D series (S2_C1D, d = 229 mm; S2_C2D, d = 226 mm; S2_C3D, d = 214 mm). Therefore, the measured values remained close to the nominal value of 215 mm, except for beams S2_C1D and S2_C2D, in which the effective depth was 14 mm and 11 mm greater, respectively.

The load–deflection curves in Figure 11 for the beams prestressed only at the bottom face (S2_T series) show that all three specimens reached similar ultimate and residual loads. However, beam S2_C2T exhibited a more pronounced stiffness loss, as cracking occurred at lower load levels than in S2_C1T and S2_C3T. As discussed in Section 4.1, this behavior was associated with the substantially lower prestressing effect in S2_C2T, due to a recorded prestress loss of 33.63%. This reduction mainly affected the service response, leading to greater flexibility, but had little influence on the ultimate condition, since the residual load was primarily governed by the mechanical capacity and effective depth of the prestressing strand.

By contrast, the load–deflection curves of the beams with symmetric prestressing on both faces (S2_D series) show a less uniform response. Two of the three beams, S2_C1D and S2_C2D, reached similar residual loads, clearly higher than that of S2_C3D. Notably, these two beams were also the ones with the greater effective depth, whereas S2_C3D, with an effective depth close to the nominal 215 mm, reached a lower residual load, more comparable to those of its counterparts in the S2_T series. Nevertheless, the comparison between S2_T and S2_D must be made with caution because the stress states prior to testing differed, as reported in Table 6. In particular, the prestressing axial force in the S2_T beams was approximately half that in the S2_D beams, while the bending moment induced by prestressing was present only in the beams prestressed at the bottom face.

#### 4.2.4. Overall Comparison Across Series

This section compares the different series by focusing on the influence of cross-sectional typology, since the effects of prestressing layout and fiber volume fraction for the three concrete types considered in the study have already been discussed in the preceding sections. To simplify the comparative analysis, only beams prestressed exclusively at the bottom face were considered. The objective is to determine, in both absolute and relative terms, which of the three typologies provides the highest efficiency and is therefore the most suitable for potential structural applications.

**Figure 14:**
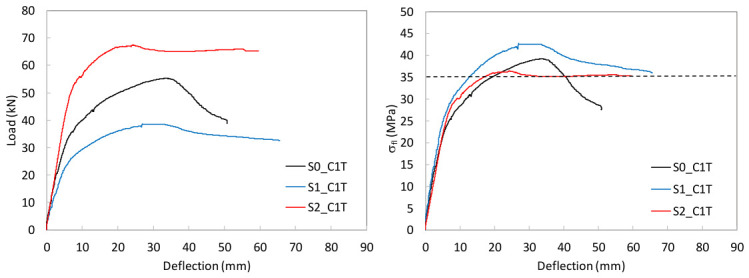
Comparison of cross-sectional typologies for C1 beams with bottom prestressing.

**Figure 15:**
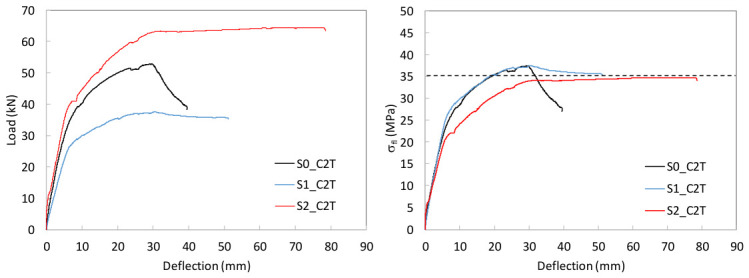
Comparison of cross-sectional typologies for C2 beams with bottom prestressing.

**Figure 16:**
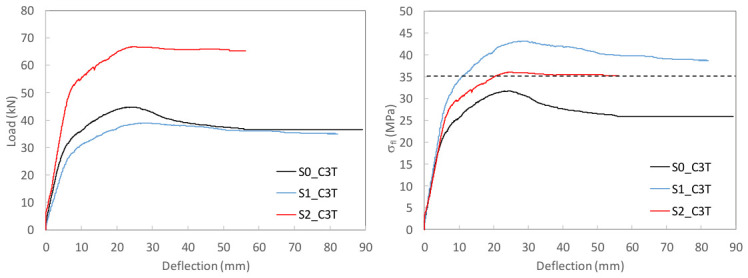
Comparison of cross-sectional typologies for C3 beams with bottom prestressing.

As shown in Figure 14, the comparison includes, on the left-hand side, the load–deflection responses of beams S0_C1T, S1_C1T, and S2_C1T, and, on the right-hand side, the equivalent flexural tensile stress–deflection curves. It should be emphasized that the equivalent flexural tensile stress (*σ_fl_*) corresponds to an equivalent stress determined under the assumption of an uncracked sectional response. This definition is consistent with the concept commonly used to characterize the equivalent flexural tensile strengths adopted in the mechanical characterization of FRCs. In the present case, *σ_fl_* was calculated using the gross moment of inertia of each cross-section (*I_gb_*), evaluated about the centroid of the gross area and assuming nominal sectional properties (S0: *I_gb_* = 0.00010139 m^4^; S1: *I_gb_* = 0.00006494  m^4^; S2: *I_gb_* = 0.00013318 m^4^), together with the distance from the centroid of the gross section to the outermost tensile fiber, taken as half of the nominal section depth (230/2 = 115 mm). Figure 15 and Figure 16 show the corresponding results for the beams made with concrete C2 (S0_C2T, S1_C2T, and S2_C2T) and concrete C3 (S0_C3T, S1_C3T, and S2_C3T), respectively.

In absolute terms, the H-shaped S2 beam showed the highest load capacity, followed by the rectangular S0 beam and, finally, the I-shaped S1 beam. This trend is consistent with the sectional configuration, since S2 is twice as wide as S0 and S1 and includes two prestressing strands instead of one. Similarly, S0 outperformed S1, as expected, since both sections share the same depth, width, and prestressing layout, with the only difference being the web reduction in S1. This reduction clearly affects the response, leading to a more pronounced post-peak drop in S0.

In relative terms, however, S1 proved to be the most efficient section, as it achieved the highest effective flexural tensile strength, followed by S0 and S2. Regarding residual behavior, S2 approached the performance of S1 at large deflections, whereas the residual branch of S0 decreased sharply. Therefore, S1 and S2 may be considered similarly efficient in terms of residual capacity, although S1 exhibited a better fiber-related response. Overall, the I-shaped S1 section, inspired by steel IPE profiles, appears to be a particularly promising solution for UHPC prestressed flexural members. In this study, equivalent flexural tensile stresses exceeding 35 MPa were achieved. These results also suggest that even higher equivalent flexural tensile stresses may be achieved by increasing the prestressing force, since the flexural capacity of the compression chord has not yet been fully exploited.

## 5. Conclusions

This experimental study investigated the bending performance and structural efficiency of eighteen slender prestressed UHPC beams without passive reinforcement, considering three cross-sectional typologies (rectangular as a reference, I-shaped, and H-shaped), three UHPC mixes with different fiber contents, and two prestressing layouts (bottom-only and symmetric top-and-bottom). Based on the results obtained, the following conclusions are drawn.

1. Slender prestressed UHPC beams with different cross-sectional typologies can be successfully produced and tested under controlled laboratory conditions. The adopted methodology, combining material characterization, strand stress monitoring, and four-point bending tests, proved suitable for interpreting their flexural response.
2. Prestress losses were influenced primarily by cross-sectional geometry and prestressing layout rather than by concrete type. The rectangular S0 series showed the lowest prestress losses, the I-shaped S1 series the highest, and the H-shaped S2 series intermediate values, except for beam S2_C2T, which showed anomalously high prestress losses due to insufficient concrete maturity at prestress transfer.
3. All beam series exhibited a similar flexural response, with an initial uncracked branch, progressive stiffness degradation after cracking, a peak load, and a residual branch associated with fiber bridging and prestressing reinforcement. No crushing of the concrete compression chord was observed at peak load, indicating that the compressive capacity of the UHPC was not fully exploited.
4. The response of I-shaped S1 beams was similar for all UHPC types (C1, C2, and C3), indicating that variations in fiber distribution and orientation do not significantly affect deflection behavior under service conditions, peak load, or residual flexural strength.
5. The maximum load recorded in bending tests of I-shaped S1 beams did not differ significantly between bottom-only and symmetric top-and-bottom prestressing, suggesting the potential of symmetric top-and-bottom prestressing arrangement for structural members experiencing both positive and negative bending moments.
6. In absolute terms, the H-shaped S2 beams reached the highest load-carrying capacity, followed by the rectangular S0 beams and the I-shaped S1 beams. In relative terms, however, the I-shaped S1 section was the most efficient typology, reaching the highest effective flexural tensile stresses and showing a favorable residual response. This highlights the strong potential of the I-shaped concept for lightweight prestressed UHPC flexural members.
7. Equivalent flexural tensile stresses above 35 MPa were achieved in the I-shaped and H-shaped elements, confirming the high structural potential of slender prestressed UHPC members. Overall, the results support their use as lightweight precast elements with competitive flexural performance. They also indicate that even higher equivalent flexural tensile stresses could be achieved by increasing the prestressing force, provided that transfer conditions and time-dependent prestress losses are properly controlled.

Future work may focus on further assessing the potential of slender prestressed UHPC members without passive longitudinal and transverse reinforcement for structural applications. Special attention should be given to achieving a more efficient use of the compressive capacity of UHPC to improve flexural performance, while preserving shear capacity and accounting for the effect of time-dependent prestress losses under higher compressive stress levels.

## Figures and Tables

**Table 1:** Cross-section details of the prestressed UHPC beams.

Beam ID	Cross-Section Shape	Fiber Volume Fraction (kg/m^3^)	Prestressing
S0_C1T	Rectangular	130 + 60	Bottom
S0_C2T	Rectangular	160	Bottom
S0_C3T	Rectangular	130	Bottom
S0_C1D	Rectangular	130 + 60	Symmetric
S0_C2D	Rectangular	160	Symmetric
S0_C3D	Rectangular	130	Symmetric
S1_C1T	I-shaped	130 + 60	Bottom
S1_C2T	I-shaped	160	Bottom
S1_C3T	I-shaped	130	Bottom
S1_C1D	I-shaped	130 + 60	Symmetric
S1_C2D	I-shaped	160	Symmetric
S1_C3D	I-shaped	130	Symmetric
S2_C1T	H-shaped	130 + 60	Bottom
S2_C2T	H-shaped	160	Bottom
S2_C3T	H-shaped	130	Bottom
S2_C1D	H-shaped	130 + 60	Symmetric
S2_C2D	H-shaped	160	Symmetric
S2_C3D	H-shaped	130	Symmetric

**Table 2:** UHPC doses considered (kg/m^3^).

Materials	Mix C1	Mix C2	Mix C3
Crush sand type 1	500	380	380
Crush sand type 2	580	700	700
Cement CEM I 42.5R SR5	800	800	800
Silica Fume	175	175	175
Dramix^®^ OL 13/0.2 Fibers	130	160	130
Dramix^®^ 3D 80/30BGP Fibers	60	-	-
Water	170	170	170
Superplasticizer	40	40	40

**Table 3:** Mechanical and physical properties of the steel fibers.

Properties	OL 13/0.2	80/30 BGP
Material	steel	steel
Fiber shape	straight	hooked-end
Bundling	loose	bundled
Length (mm)	13	30
Diameter (mm)	0.2	0.38
Aspect ratio	65	80
Tensile strength (MPa)	3050	3070
Modulus of elasticity (GPa)	210	210

**Table 4:** UHPC compressive strength of prestressed beams.

Beam ID	f_cm_ (MPa) 2 Days	f_cm_ (MPa) 7 Days	f_cm_ (MPa) 28 Days	f_cm_ (MPa) 90 Days
S0_C1T	—	103.6 (2.2%)	129.0 (3.4%)	141.7 (6.1%)
S0_C2T	96.4 (2.5%)	114.1 (6.0%)	132.2 (4.6%)	145.0 (6.1%)
S0_C3T	87.5 (7.7%)	—	131.9 (3.3%)	—
S0_C1D	92.9 (4.0%)	115.9 (6.9%)	137.9 (3.3%)	143.8 (6.6%)
S0_C2D	92.9 (1.2%)	—	141.9 (0.0%)	—
S0_C3D	90.3 (1.0%)	106.9 (2.9%)	126.6 (0.6%)	139.8 (6.0%)
S1_C1T	—	103.6 (2.2%)	129.0 (3.4%)	141.7 (6.1%)
S1_C2T	96.4 (2.5%)	114.1 (6.0%)	132.2 (4.6%)	145.0 (6.1%)
S1_C3T	87.5 (7.7%)	—	131.9 (3.3%)	143.8 (6.6%)
S1_C1D	92.9 (4.0%)	115.9 (6.9%)	137.9 (3.3%)	—
S1_C2D	92.9 (1.2%)	—	141.9 (0.0%)	—
S1_C3D	90.3 (1.0%)	106.9 (2.9%)	126.6 (0.6%)	139.8 (6.0%)
S2_C1T	82.3 (4.1%)	106.3 (2.0%)	144.0 (0.6%)	142.4 (2.0%)
S2_C2T	67.3 (3.1%)	98.7 (3.8%)	129.8 (7.0%)	144.2 (5.3%)
S2_C3T	84.4 (1.1%)	108.4 (1.4%)	132.3 (2.8%)	—
S2_C1D	82.3 (4.1%)	106.3 (2.0%)	144.0 (0.6%)	142.4 (2.0%)
S2_C2D	95.0 (0.6%)	112.9 (4.3%)	138.0 (3.0%)	—
S2_C3D	84.4 (1.1%)	108.4 (1.4%)	132.3 (2.8%)	—

— Data unavailable due to laboratory constraints, without affecting the reliability of the reported results.

**Table 5:** Evolution of prestress forces in UHPC beams.

Beam ID	Pre-Transfer Force P_1_ (kN)	P_1_/f_pu_ %	Pre-Test Force P_2_ (kN)	P_2_/f_pu_ %	(P_1_ − P_2_)/P_1_ %
S0_C1T	80.85	83.43	70.74	73.00	12.50
S0_C2T	76.36	78.80	67.61	69.77	11.46
S0_C3T	76.85	79.30	66.72	68.85	13.18
S0_C1D	150.45	78.99	136.73	71.79	9.12
S0_C2D	151.77	78.31	139.18	71.81	8.30
S0_C3D	143.64	74.11	131.10	67.64	8.73
S1_C1T	76.58	80.41	59.79	62.79	21.92
S1_C2T	74.67	77.06	58.87	60.75	21.16
S1_C3T	73.26	75.60	60.47	62.40	17.46
S1_C1D	159.15	82.12	127.87	65.98	19.65
S1_C2D	148.57	76.65	127.00	65.53	14.52
S1_C3D	148.65	76.70	126.17	65.10	15.12
S2_C1T	157.52	81.28	132.23	68.23	16.06
S2_C2T	160.02	82.56	106.21	54.80	33.63
S2_C3T	160.93	83.04	132.13	68.18	17.90
S2_C1D	297.36	78.06	250.02	65.64	15.92
S2_C2D	282.56	72.90	239.85	61.90	15.12
S2_C3D	308.78	79.66	253.17	65.31	18.01

**Table 6:** Test beam results.

Beam ID	P_u_ (kN)	δ_u_ (mm)	δ_max_ (mm)	N_p_ (kN)	M_p_ (kNm)	σ_p_ (MPa)
S0_C1T	55.26	33.55	50.85	70.74	−7.07	1357.8
S0_C2T	52.84	29.08	39.51	67.61	−6.76	1297.7
S0_C3T	44.78	24.42	89.25	66.72	−6.67	1280.6
S0_C1D	50.32	28.34	39.04	136.73	0.00	1335.3
S0_C2D	43.90	21.74	40.67	139.18	0.00	1335.7
S0_C3D	46.17	36.25	74.31	131.10	0.00	1258.1
S1_C1T	38.71	26.93	65.60	59.79	−5.98	1167.9
S1_C2T	37.50	30.04	51.20	58.87	−5.89	1130.0
S1_C3T	38.93	26.91	82.19	60.47	−6.05	1160.6
S1_C1D	40.02	34.11	81.78	127.87	0.00	1227.2
S1_C2D	37.74	26.69	48.72	127.00	0.00	1218.9
S1_C3D	38.92	33.35	61.10	126.17	0.00	1210.9
S2_C1T	67.37	24.16	59.59	132.23	−13.22	1269.1
S2_C2T	64.42	75.25	78.55	106.21	−10.62	1019.3
S2_C3T	66.78	24.55	56.28	132.13	−13.21	1268.1
S2_C1D	77.53	28.26	62.28	250.02	0.00	1220.9
S2_C2D	77.81	23.99	61.94	239.85	0.00	1151.3
S2_C3D	70.46	32.29	61.02	253.17	0.00	1214.8

## Data Availability

The original contributions presented in this study are included in the article. Further inquiries can be directed to the corresponding author.

## Abbreviations

The following abbreviations are used in this manuscript:

*σ_cp_*	concrete stress at the tendon level
*σ_fl_*	equivalent flexural tensile stress
*σ_p_*	stress in the prestressing reinforcement
4PBT	four-point bending test
*f_cm_*	average concrete compressive strength
*M_p_*	bending moment induced by prestressing
*N_p_*	axial force induced by prestressing
*P*_1_	prestressing force before transfer
*P*_2_	prestressing force before the bending test
*P_u_*	ultimate load attained during the bending test
UHPC	ultra-high-performance concrete
UHPFRC	ultra-high-performance fiber-reinforced concrete
VWSG	vibrating-wire strain gauges
*δ_max_*	maximum deflection attained in the bending test
*δ_u_*	deflection at mid-span attained at ultimate load in the bending test
